# Unraveling the Biopsychosocial Factors of Fatigue and Sleep Problems After Traumatic Brain Injury: Protocol for a Multicenter Longitudinal Cohort Study

**DOI:** 10.2196/11295

**Published:** 2018-10-22

**Authors:** Jessica Bruijel, Sven Z Stapert, Annemiek Vermeeren, Jennie L Ponsford, Caroline M van Heugten

**Affiliations:** 1 Department of Neuropsychology & Psychopharmacology Faculty of Psychology and Neuroscience Maastricht University Maastricht Netherlands; 2 Limburg Brain Injury Centre Maastricht Netherlands; 3 Department of Clinical and Medical Psychology Zuyderland Medical Centre Sittard-Geleen Netherlands; 4 School of Psychological Sciences and Monash Institute of Cognitive and Clinical Neurosciences Monash University Melbourne Australia; 5 Monash-Epworth Rehabilitation Research Centre Epworth Healthcare Melbourne Australia; 6 School for Mental Health and Neuroscience Department of Psychiatry and Neuropsychology, Faculty of Health, Medicine and Life Sciences Maastricht University Medical center Maastricht Netherlands

**Keywords:** traumatic brain injury, sleep, fatigue, biopsychosocial model

## Abstract

**Background:**

Fatigue and sleep problems are common after a traumatic brain injury (TBI) and are experienced as highly distressing symptoms, playing a significant role in the recovery trajectory, and they can drastically impact the quality of life and societal participation of the patient and their family and friends. However, the etiology and development of these symptoms are still uncertain.

**Objective:**

The aim of this study is to examine the development of fatigue and sleep problems following moderate to severe TBI and to explore the changes in underlying biological (pain, brain damage), psychological (emotional state), and social (support family, participation) factors across time.

**Methods:**

This study is a longitudinal multicenter observational cohort study with 4 measurement points (3, 6, 12, and 18 months postinjury) including subjective questionnaires and cognitive tasks, preceded by 7 nights of actigraphy combined with a sleep diary. Recruitment of 137 moderate to severe TBI patients presenting at emergency and neurology departments or rehabilitation centers across the Netherlands is anticipated. The evolution of fatigue and sleep problems following TBI and their association with possible underlying biological (pain, brain damage), psychological (emotional state), and social (support family, participation) factors will be examined.

**Results:**

Recruitment of participants for this longitudinal cohort study started in October 2017, and the enrollment of participants is ongoing. The first results are expected at the end of 2020.

**Conclusions:**

To the authors’ knowledge, this is the first study that examines the development of both post-TBI fatigue and sleep longitudinally within a biopsychosocial model in moderate to severe TBI using both subjective and objective measures. Identification of modifiable factors such as mood and psychosocial stressors may give direction to the development of interventions for fatigue and sleep problems post-TBI.

**Trial Registration:**

Netherlands Trial Register NTR7162; http://www.trialregister.nl/trialreg/admin/rctview.asp?TC=7162 (Archived by WebCite at http://www.webcitation.org/6z3mvNLuy)

**International Registered Report Identifier (IRRID):**

RR1-10.2196/11295

## Introduction

Traumatic brain injury (TBI) is one of the most serious, disabling neurological disorders, with 10 million patients affected annually worldwide [1]. Consequently, societal costs are high and estimated to be around €33 billion in Europe [2]. TBIs appear on a spectrum of injury severity based on widely recognized injury characteristics. The more frequent mild TBIs are considered as trivial and benign injuries as opposed to less prevalent moderate to severe injuries, which are associated with long-lasting consequences for the patients and their environment [3]. Due to the high individual and societal costs associated with extensive rehabilitation needs and chronic disability, moderate to severe TBI represents a critical public health issue [4], with fatigue and sleep problems playing significant roles in the recovery process [5,6]. Between 30% and 70% of the patients experience fatigue [7], and a meta-analysis indicated that 53% experience sleep problems [8].

Study results concerning the presence of fatigue and type of sleep problems post-TBI are inconsistent, probably due to different study methodologies. Patients are included at different time points since their injuries and injury severity parameters differ across studies, measurement instruments are diverse, and there is limited consensus on what variables at which moment in time should be measured [9]. In addition, most studies are cross-sectional and not longitudinal in terms of design. This makes it difficult to compare results across studies and to draw conclusions about sleep and fatigue changes after TBI [10,11]. Nevertheless, post-TBI sleep problems and fatigue are often consistently experienced as the most severe and distressing symptoms [5], interfering with recovery and rehabilitation treatment and negatively impacting the quality of life [12]. Furthermore, despite the magnitude and impact of these phenomena, the etiology is still debated and no efficacious treatments have been established [13].

Recovery from moderate to severe TBI is a time-consuming and long-term process and should, therefore, be explained in terms of a disease process. Accordingly, different factors may be involved in fatigue and sleep problems at different stages after the injury [14,15]. By exploring the underlying causes of fatigue and sleep problems and how these symptoms develop over time, key periods may be identified in which specific targeted interventions are needed. The outcome and prognosis following TBI are extremely variable across individuals regardless of the severity of the initial injury [9], which implies that outcome is not only influenced by biological factors but should be studied in a biopsychosocial model in which physical, cognitive, affective, and social factors interact with sleep-wake patterns and fatigue [7,9,16,17]. Previous research has already shown the involvement of biological factors (eg, structural changes in the brain [18] and pain [19]) and psychological (eg, emotional distress [20,21]) and social components (eg, community integration and social support [22,23]) in fatigue and sleep problems following TBI. These factors are also involved in sleep and fatigue in other chronic diseases such as cancer, multiple sclerosis, and diabetes [24]. However, no studies, to the authors’ knowledge, have yet examined these biopsychosocial factors in a comprehensive model over time to determine the significant underlying factors that contribute to post-TBI fatigue and sleep problems. Understanding these complex interactions is crucial to establish, explain, and treat fatigue and sleep problems associated with TBI. Therefore, this study proposes a biopsychosocial explanation of post-TBI fatigue and sleep problems.

The aim of the study is to examine the development of post-TBI fatigue and sleep problems longitudinally within a biopsychosocial model including several factors in moderate to severe TBI. The primary focus of the study will be on subjective fatigue and sleep problems post-TBI. We hypothesize that the associations between biopsychosocial factors and post-TBI fatigue and sleep problems change over time, that is, the associations with biological factors are strongest in the first 6 months and then decline, whereas the associations with psychological and social factors are initially weak but slowly increase and become apparent between 12 and 18 months. Previous research has shown a discrepancy between objective and subjective measures of fatigue and sleep in the TBI population [21,25]. Therefore, the secondary aim of the study is to examine the development of post-TBI fatigue and sleep problems with objective measures within a biopsychosocial model. In this paper, the design of the study will be presented.

## Methods

### Design

This study is a multicenter, observational, prospective longitudinal cohort study in which participants are followed using 5 assessments during the first 18 months following moderate to severe TBI. The Medical Ethics Committee of University Hospital Maastricht/Maastricht University (NL60322.068.17) and all participating centers approved the study protocol. The study is registered in the Dutch Trial Register (NTR67162, registered on April 10, 2018).

### Study Population

Moderate to severe TBI patients are being recruited from emergency, neurology, and rehabilitation departments in several hospitals and rehabilitation clinics across the Netherlands. On the basis of a linear mixed regression analysis with a medium effect size (*F*_*2*_=0.15), 7 significant predictors, a statistical power of 0.8, alpha level of .05, and a high test-retest reliability of at least 0.8 of the main study variables, the required sample is 103 TBI patients [26]. A dropout of 25% during the 18-month follow-up is expected based on previous studies [27,28]. Therefore, 137 patients will be recruited to lead to a total of 103 TBI patients being available for the analyses.

### Inclusion and Exclusion Criteria

TBI patients are eligible to participate in this study if they have a clinically confirmed diagnosis of a first moderate to severe, closed-head TBI, which is defined as Glasgow Coma Scale score<13 [29]; post-traumatic amnesia (PTA)>24 hours; trauma-related intracranial neuroimaging abnormalities; or loss of consciousness (LOC)>30 min [30]. In addition, participants must be aged between 21 and 70 years, fluent in Dutch, and provide informed consent.

Participants are excluded if they (1) had a prior moderate to severe TBI diagnosed by a neurologist or a mild concussion in the last half year; (2) have another condition that may interfere with the study outcome (eg, other pre-existing neurological disorder [stroke, brain tumor, etc], sleep-wake disturbance, fatigue due to any medical condition other than TBI, history of alcohol or drug abuse, prior mental disorder [for which treatment was necessary], or pregnancy); or (3) lack the ability to complete questionnaires based on clinical judgment (aphasia, severe cognitive impairment).

Participants meeting the following criteria are excluded during the study: (1) participant wants to leave the study or (2) there is a new incidence of TBI, other neurological disease/injury, or traumatic injury during the follow-up period.

### Procedure

Patients are informed about the study by their treating physician (eg, neurologist, head nurse, or rehabilitation specialist). If the patient is interested in participating, a screening visit within the first 6 weeks after injury is done by the researcher, during which the informed consent is signed (if the patient is eligible and decides to participate). During this visit, demographics and preinjury characteristics are collected.

The follow-up appointments take place at approximately 3 months (V1), 6 months (V2), 12 months (V3), and 18 months (V4) postinjury, within 2 weeks before or after the exact follow-up date (ie, time window of 1 month). These visits consist of filling out questionnaires and performing cognitive tasks and can take place at Maastricht University, one of the participating clinical institutes, or the home of the participant. The visit will be guided by the researcher or a research assistant and are always scheduled between 11:00 am and 3:00 pm to minimize effects of the circadian rhythm [31]. In the week before these visits, the participant will wear an actigraph and fill out a sleep diary for 7 days at home (daily living). A reminder phone call is given at the start of the registration period, and during the 7 days, we will phone the participants twice to remind them. With the participant’s permission, partners or family members of the participant are informed about the study to monitor whether the actigraph is worn. Participants receive 10 euros for each follow-up visit, and their travel expenses are reimbursed.

### Measurements

The main outcomes are fatigue and sleep. The primary focus of this study is on subjective level of fatigue and sleep problems, affecting the quality of sleep, to address the experience of these problems by TBI patients. The relation over time between subjective fatigue and sleep and the biopsychosocial predictors shown in Table 1 will be examined. Second, the relation between objective fatigue and sleep measurements and the biopsychosocial predictors will be examined. An overview of all measurement instruments that are administered during the 18-month follow-up is shown in Table 1. The questionnaires are implemented in an online format, except for the demographic questionnaire, which is in an interview style. All questionnaires included in this study have good psychometric properties and have been used in the TBI population before.

#### Primary Outcome Measures

Subjective fatigue is measured with the Fatigue Severity Scale (FSS) [32]. The FSS is widely used, and it measures the impact of fatigue on activities of daily life and distress caused by fatigue; it includes 9 items related to fatigue, which are rated on a 7-point Likert scale. The mean score of the FSS is calculated and ranges from 1 to 7, where a higher score denotes more severe fatigue and a mean score of 4 or higher indicates severe fatigue [32]. The internal consistency is high [32], test-retest reliability is satisfactory, and the FSS can distinguish fatigue in brain-injured patients from that of controls [49].

Subjective sleep quality is assessed with the Pittsburgh Sleep Quality Index (PSQI) [33]. The PSQI consists of 19 items and examines 7 components, namely, overall sleep quality, sleep onset latency, total sleep time, sleep efficiency, sleep disturbances, use of sleep medication, and daytime dysfunction. The global score is calculated by adding the 7 component scores and ranges from 0 to 21, where a lower score denotes better sleep quality. The questionnaire can discriminate between “good” and “poor” sleepers, with a global score of >5 indicating poor sleep quality [33]. The internal consistency and test-retest reliability of the PSQI are high, and the PSQI has good concurrent validity with sleep diary data [33]. The Dutch version of the PSQI has been used to examine sleep quality in acquired brain injury patients [50].

#### Predictors

The development of sleep and fatigue is examined with a biopsychosocial model. Therefore, the factors taken into account as predictors can be divided in biological (eg, structural changes in the brain and pain), psychological (eg, emotional distress and the burden of cognitive impairments), and social components (eg, community integration and social support).

##### Pain

The general level of pain is measured with a 100-mm visual analog scale [34]. The left end of the VAS represented “no pain” and the right end represented “most severe pain imaginable” with no intermediate divisions or descriptive terms [34]. The score ranges from 0 to 10, where a higher score indicates more severe pain. Pain intensity in the last 24 hours is measured. The VAS is widely used to measure pain in TBI patients [22], and it is suggested as a valid and reliable measure [51].

**Table 1:** Overview of all measurement instruments for the traumatic brain injury patients and the times of administration.

Parameter		Instrument	Screening (<6 weeks)	3 months	6 months	12 months	18 months
**Main outcome parameters**							
	Subjective fatigue	Fatigue Severity Scale [32]	—	✓	✓	✓	✓
	Subjective sleep quality	Pittsburgh Sleep Quality Index [33]	—	✓	✓	✓	✓
**Predictors**							
	Pain (subjective)	Visual analogue scale pain [34]	—	✓	✓	✓	✓
	Objective cognitive performance	Stroop, COWAT^a^, digit span, SDMT^b^ [35]	—	✓	✓	✓	✓
	Physical activity	7 days actigraphy [36]	—	✓	✓	✓	✓
	Emotional distress	Hospital anxiety and depression scale [37]	—	✓	✓	✓	✓
	Cognitive complaints	Dysexecutive Questionnaire Revised [38]	—	✓	✓	✓	✓
	Participation	Utrecht Scale for Evaluation and Rehabilitation-Participation [39]	—	✓	✓	✓	✓
	Social support	Multidimensional Scale of Perceived Social Support [40]	—	✓	✓	✓	✓
**Secondary outcome parameters**							
	Objective sleep wake disturbances	7-days actigraphy [41]	—	✓	✓	✓	✓
	Objective fatigue	Psychomotor vigilance task [42]	—	✓	✓	✓	✓
**Group characteristics and monitor the participants**							
	TBI characteristics	Injury severity such as structural imaging data, LOC^c^, PTA^d^, injury severity score; causes of injury; comorbid (physical) injuries, seizures; drug or alcohol intoxication during injury from the hospital database.	✓	—	—	—	—
	Demographics	Age, gender, education, marital status, and work status	✓	—	—	—	—
	Premorbid sleep	Premorbid question of PSQI^e^ [33]	✓	—	—	—	—
	Premorbid participation	Premorbid frequency and satisfaction of the USER-P^f^ [39]	✓	—	—	—	—
	Daytime sleepiness	Epworth Sleepiness Scale [43]	—	✓	✓	✓	✓
	Multidimensional aspects of fatigue	Dutch Multi-Factor Fatigue Scale [44]	—	✓	✓	✓	✓
	Subjective sleep-wake	7 days sleep diary [45]	—	✓	✓	✓	✓
	Posttraumatic stress disorders	PTSD^g^ checklist for DSM-5 [46]	—	—	✓	—	✓
	Coping style	Proactive and passive coping scale of the Utrecht Coping List [47]	—	✓	—	—	✓
	Drugs/alcohol/medication use	Demographic questionnaire	✓	✓	✓	✓	✓
	Sleepiness preceding the task	Karolinska sleepiness scale [48]	—	✓	✓	✓	✓

^a^COWAT: controlled word association test.

^b^SDMT: symbol digit modalities test.

^c^LOC: loss of consciousness.

^d^PTA: posttraumatic amnesia.

^e^PSQI: Pittsburgh Sleep Quality Index.

^f^USER-P: Utrecht Scale for Evaluation and Rehabilitation-Participation.

^g^PTSD: posttraumatic stress disorder.

##### Cognition

A short test battery is used to assess cognitive performance. The extent to which cognitive functioning is affected is used as a proxy for the severity of the brain damage [52]. Cognitive tasks include measurements of speed, attention, interference, and executive functioning. The following 4 tasks are included, and the first 3 tasks are recommended as outcome measures in TBI research to measure neuropsychological impairments [35]:

1. *Stroop task* measures response interference control, a cognitive form of inhibition/flexibility and selective attention [53]. Previous studies showed inhibition deficits following TBI and a slower response time [54]. The stroop has good psychometric properties [35].
2. *Controlled oral word association test* (COWAT) [55] is a verbal fluency test, which measures the spontaneous production of words belonging to a specific category or a designated letter. This test measures attentional control, working memory, and other components of executive functioning. Focal frontal injuries following TBI show a strong association with performance on the COWAT [56]. COWAT is a reliable measure and is sensitive to TBI severity [57].
3. *Digit span* is a working memory task that assesses auditory attention. Both the forward and the backward order are used. The digit backward order is especially informative for working memory. This task has been used as a marker of cognitive deficit and recovery and has a high reliability [57].
4. *Symbol digit modalities test* (SDMT) is a cognitive test that measures attention and processing speed. The SDMT is sensitive to impairments of speed of information processing following TBI [58] and is a reliable measure [59].

##### Physical Activity

Daytime levels of physical activity are examined with actigraphy, which is a noninvasive method to monitor the rest/activity cycle [36]. In addition, actigraphy is used for the secondary aim regarding objective measures of sleep. The actigraph is a wristwatch-like device, worn on the nondominant wrist, which allows the participant to continue normal routines in the natural environment. There is no remote monitoring whether the actigraph is worn; however, the actigraph can be worn continuously during this week also when bathing. The actigraph (GENEActiv, Activinsights Ltd, Cambridgeshire, United Kingdom) measures the movement/motor activity of the participant, and thereby, the time spent in sedentary behavior, light intensity physical activity, moderate to vigorous physical activity, and vigorous physical activity can be determined [36]. Participants will wear the actigraph for 1 week.

##### Emotional Distress

The level of emotional distress is examined with the Hospital Anxiety and Depression Scale (HADS) [37], which consists of 14 items. Each item is scored on a 4-point scale, and the total score ranges from 0 to 42, where a higher score denotes more psychological distress. The HADS includes 2 subscales with each 7 items measuring anxiety and depression with scores ranging from 0 to 21. A subscale score of **≥**8 is an indicator of depression or anxiety in patients with TBI, which is in line with findings of the general population [60]. The HADS is a reliable measure and has been validated in the TBI population [61].

##### Cognitive Complaints

The Dysexecutive Questionnaire Revised (DEX-R) is used to assess cognitive complaints [38]. This questionnaire examines cognitive problems in daily life as experienced by the patient. The DEX-R assesses 4 domain-general types of dysexecutive problems (metacognition or social cognition, executive cognition, behavioral-emotional self-regulation, and activation) and comprises 34 items. Each item is scored on a 5-point Likert scale on how often certain difficulties related to cognition are experienced. The total score ranges from 0 to 136, where a higher score denotes more cognitive problems. The DEX-R is a reliable and valid measure [38,62] and has been used in the TBI population [63].

##### Participation

The Utrecht Scale for Evaluation and Rehabilitation-Participation (USER-P) [39] is used to assess participation. The questionnaire measures 3 aspects of participation: frequency of behaviors, experienced participation restrictions due to health condition, and satisfaction with participation. The USER-P consists of 31 items across the 3 subscales. Each sum score of a scale is converted to scores ranging from 0 to 100, where higher scores indicate good levels of participation (higher frequency, fewer restrictions, higher satisfaction). The USER-P is a valid and reliable measure in patients with brain injury, and test-retest reliability and internal consistency of the USER-P are satisfactory [64].

##### Social Support

The Multidimensional Scale of Perceived Social Support (MSPSS) is used to assess social support [40]. The MSPSS consists of 12-items examining perceived social support from family, friends, and significant other. Each item is rated on a 7-point Likert scale. The mean total score ranges from 1 to 7, where a higher score denotes more perceived social support. The MSPSS has shown good psychometric properties [40], and it has been used in TBI patients [65].

#### Secondary Outcome Measures

Previous research has shown a discrepancy between objective and subjective measures of fatigue and sleep in TBI population [21,25]. Therefore, as the secondary aim, objective measures of fatigue and sleep are included in this study.

Fatigue is measured objectively with the 10-min psychomotor vigilance task (PVT), which is a sustained-attention, reaction-time task, often used in sleep and fatigue research [42]. The PVT is a simple, reliable, and sensitive task for measuring performance and attentional deficits due to fatigue [66]. When performing the PVT, the response time to visual stimuli occurring at random interstimulus intervals is measured. The task has good psychometric properties, has been validated, and has been used in TBI patients [67].

Sleep problems are examined objectively with the actigraph described previously that measures sleep-wake patterns during 1 week. Actigraphy has shown to be a satisfactory objective estimate of sleep especially for global sleep parameters including total sleep time, sleep onset latency, and sleep efficiency [41]. Multiple studies have included actigraphy to examine sleep in TBI patients [25,68,69], and they have shown that actigraphy is a reliable method for monitoring sleep in this population, irrespective of the injury severity [70].

#### Group Characteristics and Monitoring Participants

##### Injury-Related Characteristics

Information regarding the injury such as time since injury, injury severity parameters (eg, intracerebral abnormality on structural imaging data, LOC, PTA, injury severity score), causes of injury, comorbid (physical) injuries, seizures, and drug or alcohol intoxication during injury will be retrieved from the hospital database.

##### Demographics

The demographic questionnaire asks about age, gender, education, marital status, level of occupational achievement, psychological, and medical history. In addition, this questionnaire assesses medication, drugs, and alcohol use.

##### Daytime Sleepiness

The Epworth Sleepiness Scale (ESS) is used to examine daytime sleepiness [43]. The ESS measures general level of daytime sleepiness and sleep propensity with 8 items. Each item is scored on a 4-point scale indicating the chance of dozing off, and the total score ranges from 0 to 24, where a higher score indicates more daytime sleepiness. A score of **≥**11 indicates clinically significant subjective sleepiness [43]. The ESS is widely used in TBI research [71] and has a reasonably high reliability [72].

##### Multidimensional Aspects of Fatigue

The Dutch Multi-Factor Fatigue Scale (DMFS) is used to measure the multidimensional aspects of fatigue. The DMFS is a newly developed questionnaire that examines several factors of fatigue following TBI, including impact of fatigue, mental fatigue, signs and direct consequences of fatigue, physical fatigue, and coping with fatigue [44]. The DMFS consists of 38 items rated on a 5-point Likert scale, with higher scores on each subscale indicating more severe fatigue. This questionnaire is specifically developed to measure the multiple facets of fatigue following acquired brain injury [44].

##### Subjective Sleep-Wake Patterns

The relevant questions of the consensus sleep diary, which is a standardized sleep diary developed by insomnia experts [45], are used to examine subjective sleep-wake patterns and for better interpretation of actigraphy data. The sleep diary includes the following core questions: (1) the time of getting into bed; (2) the time at which the individual attempted to fall asleep; (3) sleep-onset latency; (4) duration of awakenings; (5) time of final awakening; (6) final rise time; and (7) perceived sleep quality (rated via Likert scale) [45]. An additional question about napping/dozing is added. The diary is completed in the morning and is filled out for 7 consecutive days concurrent with the actigraphy. Sleep diaries are a reliable and validated measure to examine sleep [73].

##### Posttraumatic Stress Disorder

The presence of posttraumatic stress disorder (PTSD) is determined with the PTSD Checklist of the Diagnostic and Statistical Manual of Mental Disorders, fifth edition (DSM-5; PCL-5), a 20-item self-reported measure corresponding to the DSM-5 symptom criteria for PTSD [46]. Each item is rated on a 5-point scale Likert scale and the total score ranges from 0 to 80, where a higher score denotes more severe PTSD symptoms. A score of 33 or higher is suggested as the indication of PTSD [46]. The PCL-5 is a reliable measure with strong validity [74]. PTSD occurs in 18% to 27% of the cases following severe TBI [75,76]. To check whether PTSD is the underlying cause of elevated stress and as PTSD takes time to develop, the PCL-5 is only assessed at visits 2 and 4.

##### Coping Style

Passive reaction coping style and active problem-solving coping style are examined with the Utrechtse Coping Lijst (UCL), which will differentiate active approach versus passive approach [47]. As this study only includes active and passive coping, the questionnaire will consist of 14 items scored on a 4-point Likert scale. Scores for both subscales range from 7 to 28, where higher scores denote a higher preference for that coping style. Both subscales show fairly good internal consistency and reasonably high test-retest reliability in the Dutch population [77]. The UCL has been used in Dutch TBI patients before and showed limited variability over time, therefore, coping styles are only assessed at visits 1 and 4 [78].

##### Sleepiness Preceding the Task

Sleepiness before the PVT is assessed with the Karolinska sleepiness scale (KSS) [48]. The KSS consist of 1 item on a 9-point Likert scale ranging from extremely alert to very sleepy, great effort to keep awake, where a higher score denotes greater sleepiness. The subject indicates the sleepiness level of the preceding 5 min. The test-retest reliability and the construct validity of the KSS are high [79].

#### Statistical Analyses

Descriptive statistics will be used to present mean scores and SDs at each time point of the outcome measures and predictive variables. Normality and assumptions will be checked. Next, 2 linear mixed regression analysis [80] will be performed to evaluate the associations between the predictive (independent) variables (pain, cognitive impairment, physical activity, emotional distress, cognitive complaints, social support, and participation) and the primary end point (subjective sleep quality and fatigue) across time. For each of the 2 primary end points, we will first determine whether these associations with predictors change across the 4 time points (ie, time by predictor interactions). In case of a significant interaction, simple interaction contrasts comparing consecutive time points will be used to determine whether the association between predictor and primary end point decreases or increases. Bonferroni correction will be used to adjust for multiple testing.

For the secondary objectives, the temporal relation between objective fatigue, objective sleep, and the predictive variables of the biopsychosocial model will be examined with the same linear mixed-effects regression analyses as used for the primary objectives.

## Results

Recruitment of participants for this longitudinal cohort study started in October 2017, and the enrollment of participants is ongoing. The first results are expected at the end of 2020.

## Discussion

This study describes the protocol of a longitudinal cohort study examining fatigue and sleep following moderate to severe TBI and the underlying predictors with a biopsychosocial model.

There are several reasons why this cohort study is innovative. First, this study has a longitudinal design. To the authors’ knowledge, there are only 3 longitudinal follow-up studies examining fatigue or sleep following moderate to severe TBI in the first 12 to 24 months post-TBI [22,81,82]. These studies had a much smaller sample size and focused on fatigue or sleep separately.

Second, even though fatigue and sleep are closely related, they can be affected independently, and problems with fatigue and sleep do not always co-occur [15]. Therefore, this study examines fatigue and sleep concurrently in a follow-up design to better understand their common and unique manifestations, as was also recommend by Cantor et al [15].

Third, this study uses a biopsychosocial explanation of post-TBI fatigue and sleep problems [9]. Multiple researchers suggested integrated biopsychosocial approaches for future studies to best explain the outcome of TBI [83-86]. However, few studies have yet examined multiple identified biopsychosocial factors in a comprehensive model over time to determine the significant underlying factors that contribute to post-TBI fatigue and sleep problems. Understanding these complex interactions is crucial to establish, explain, and treat fatigue and sleep problems associated with TBI.

Finally, this study uses both subjective and objective measures to examine fatigue and sleep. Previous research has shown discrepancies between objective and subjective measures of fatigue and sleep in the TBI population [21,25]. Therefore, it is important to include both measures. However, most studies only include subjective or objective measures of fatigue and sleep.

A limitation of this study is that the extreme, severe multitrauma patients will not be included in the study because they may not be recognized as TBI due to severe multiple physical injuries and they may not be able to participate due to their injuries. This may jeopardize the generalizability of the results to all moderate to severe TBI patients.

To the authors’ knowledge, this study will be the first that examines the development of both post-TBI fatigue and sleep longitudinally with a biopsychosocial model in moderate to severe TBI and that will differentiate between fatigue and sleep using both subjective and objective measures. Identification of modifiable factors such as mood and psychosocial stressors may give direction to the development of interventions for fatigue and sleep problems post-TBI that subsequently lower the burden for the patient and may prevent the development of secondary symptoms and complaints such as depression.

## Abbreviations

COWAT: controlled oral word association test
DMFS: Dutch Multi-Factor Fatigue Scale
DEX-R: Dysexecutive Questionnaire Revised
ESS: Epworth Sleepiness Scale
FSS: Fatigue Severity Scale
HADS: Hospital Anxiety and Depression Scale
KSS: Karolinska sleepiness scale
LOC: loss of consciousness
MSPSS: Multidimensional Scale of Perceived Social Support
PCL-5: PTSD Checklist for DSM-5
PSQI: Pittsburgh Sleep Quality Index
PTA: posttraumatic amnesia
PTSD: posttraumatic stress disorder
PVT: psychomotor vigilance test
SDMT: symbol digit modalities test
TBI: traumatic brain injury
UCL: Utrechtse Coping Lijst
USER-P: The Utrecht Scale for Evaluation of Rehabilitation-Participation (In Dutch: Utrechtse Schaal voor Evaluatie van Participatie)
